# Identifying false-positive drug allergy alerts based on drug tolerance assertions: a retrospective study

**DOI:** 10.1093/jamiaopen/ooag010

**Published:** 2026-01-27

**Authors:** Jakir Hossain Bhuiyan Masud, James J Cimino, Tiago K Colicchio

**Affiliations:** Department of Biomedical Informatics and Data Science, University of Alabama at Birmingham, Birmingham, AL 35294, United States; Department of Biomedical Informatics and Data Science, University of Alabama at Birmingham, Birmingham, AL 35294, United States; Department of Biomedical Informatics and Data Science, University of Alabama at Birmingham, Birmingham, AL 35294, United States

**Keywords:** electronic health records, clinical decision support, drug allergy, alert fatigue

## Abstract

**Objective:**

This study aimed to evaluate the characteristics of drug allergy alerts (DAAs) and identify false-positive alerts by analyzing prior drug administration records.

**Materials and Methods:**

We retrospectively analyzed DAAs fired to providers with prescribing authority in 2023 at a large academic medical center in the Southeast to identify data elements that could help reduce clinically irrelevant alerts.

**Results:**

Overall, 101 492 DAAs were triggered in 2023, with a 98.9% override rate. Alerts were fired for 9111 unique patients (an average of 11.1 alerts per patient). Only 9.7% DAAs had a definite match between the prescribed drug and documented allergen, with the remaining 90.3% fired for different drugs under the same class or allergen group. Overall, 70% DAAs were triggered for patients with prior administration of the drug triggering the alert, of these, 74% (52% of all DDAs) occurred in patients who had received the prescribed drug after initial allergy documentation, and 79% (56% of all DAAs) received the same drug again after 2023. Patients who had received prescribed drug previously, definite match were more likely to be overridden than no match (OR = 1.18, 95% CI: 1.03-1.33, *P* = .013) with a slightly higher override rate (98.9%% [*n* = 71 357] vs 98.8% [*n* = 30 135]).

**Discussion:**

Most DAAs occurred in patients previously exposed to the alerting medication, often after allergy documentation, and over half of the patients continued to receive the same drug after 2023.

**Conclusion:**

Future research should focus on examining strategies to incorporate tolerance assertions into DAA logic to reduce false-positives without compromising safety.

## Background and significance

Over the past decade, electronic health records (EHRs) have been widely adopted across the U.S. health system, largely driven by commercial systems adopted with infusion of tens of billions of federal stimulus dollars through the Meaningful Use program.^1^^,^^2^ Although useful for workflow integration, and data storage and processing, these systems were adopted without improvements needed to address key limitations, resulting in unintended consequences at multiple care levels.^3^ As a noticeable example, clinical decision support (CDS) systems in the form of alerts fired at the point of care have been widely adopted to improve decision-making, reduce errors, and enhance patient safety^4–6^; yet, the same systems often lead to disruptions to clinical workflow with excessive, overzealous alerts that are frequently ignored by providers, and contribute to the so called “alert fatigue.”^7^ Over time, false-positive alerts can lead a form of automatism where alert override becomes a habitual behavior,^8^ causing clinicians to overlook true-positive alerts with real potential to prevent harm.^3^

Alert fatigue is commonly reported in the context of medication-related alerts.^9–12^ While CDS systems have been associated with reducing medication errors by as much as 81% in inpatient settings,^13^^,^^14^ their effectiveness is off-set by high override rates that can rise above 90%.^15^^,^^16^ One type of CDS alert that is often subject to high override rates is the drug allergy alert (DAA), which are often found to be among the top most frequently triggered (and overridden) alerts in hospital settings.^17^^,^^18^ Although many overrides are clinically justified, inappropriate overrides pose significant risks, including a 6-fold increase in the risk of adverse drug events (ADEs).^19^ This is particularly concerning due to studies showing that inappropriate overrides are often observed even when the documented reaction is life-threatening (eg, anaphylaxis).^20^

Despite these challenges, well-designed CDS tools have the potential to improve patient outcomes. Adherence to the “Five Rights” framework—delivering the right information to the right person, in the right format, through the right channel, and at the right time—remains critical for effective CDS implementations.^21^ Efforts to address these challenges have focused on enhancing the specificity and sensitivity of alerts, with strategies primarily focused on refining alert logic, tailoring alerts to individual user preferences, and prioritizing alerts based on their clinical significance.^22^^,^^23^ These strategies often involve refinement of CDS logic or retirement of irrelevant alerts by expert panels.^24^ In the context of DAAs, some researchers have attempted to improve allergy documentation with manual and semi-automated methods but have achieved only moderate results,^25–27^ while others have measured the incidence of prior drug administration in patients with allergy to penicillin, which represented only 8% of all DAAs.^28^

In a prior study, we measured the incidence of overridden DAAs in the calendar year of 2019 in patients with allergy to opioids.^29^ We examined the incidence of DAAs triggered by low-risk cross-sensitivity opioid pairs or in patients with prior administration of the prescribed opioid, which combined accounted for 46.4% of all DAAs at our organization. While this preliminary study^29^ revealed that prior administration of a drug triggering an alert was associated with higher override rate, inclusion of a single drug class limited external validity. In the present study, we expand and update our analysis using data from 2023 and analyze override rate with and without prior drug administrations for all drug classes.

## Objective

This is a retrospective observational study to examine DAAs fired and overridden at a large academic medical center. Based on commonly reported override reasons and DAA appropriateness identified by Luri et al,^30^ we formulated the following research questions:

1. What are the characteristics of DAAs at our institution, including the volume of alerts fired, override rates, documented allergic reactions, and override reasons?
2. Is there an association between alert status (eg, accepted, overridden) and the documentation of prior drug administrations?

Our objective is to analyze the characteristics of DAAs at our institution and identify those that are false-positive, based the patient’s prior drug administrations.

## Materials and methods

### Settings and participants

This study was conducted at the University of Alabama at Birmingham (UAB) Hospital, a 1306-bed tertiary care facility and academic medical center located in Birmingham, Alabama, with long-standing history of developing and implementing informatics applications.^31^ The hospital’s current EHR system is Millennium (Oracle Cerner, Kansas City, MO). The EHR provides a DAA functionality designed to notify users of potential allergic reactions based three types of match:

1. Definite Match: This occurs when there is an exact match between the triggering drug (ie, the drug being prescribed when an alert is fired) and the allergen documented in the patient’s allergy history. For example, if the patient’s allergy list includes penicillin and the prescribed drug is also penicillin, a definite match DAA will be triggered.
2. Probable Match: This alert is triggered when the triggering drug and the documented allergen differ but are under the same drug class in the Cerner Multum Drug Database. (eg, prescribed drug = codeine, documented allergen = morphine). Note: drug classes used in the comparisons were defined using the original Multum Drug Database integrated into Oracle Cerner.
3. Possible Match (Cross-Sensitivity): This type of alert is triggered when the triggering drug and the documented allergen differ but are under the same allergen group in Cerner Multum (eg, prescribed drug = furosemide, documented allergen = bactrim).

**Figure 1.**
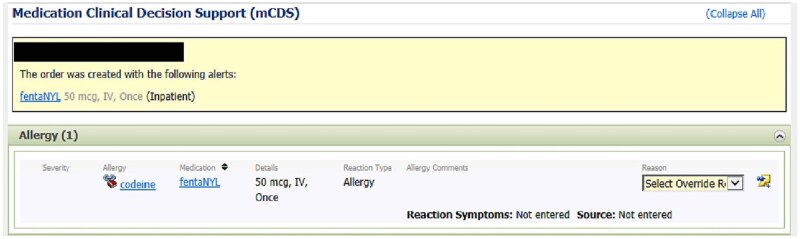
Example of a DAA fired in our EHR system.

**Figure 2.**
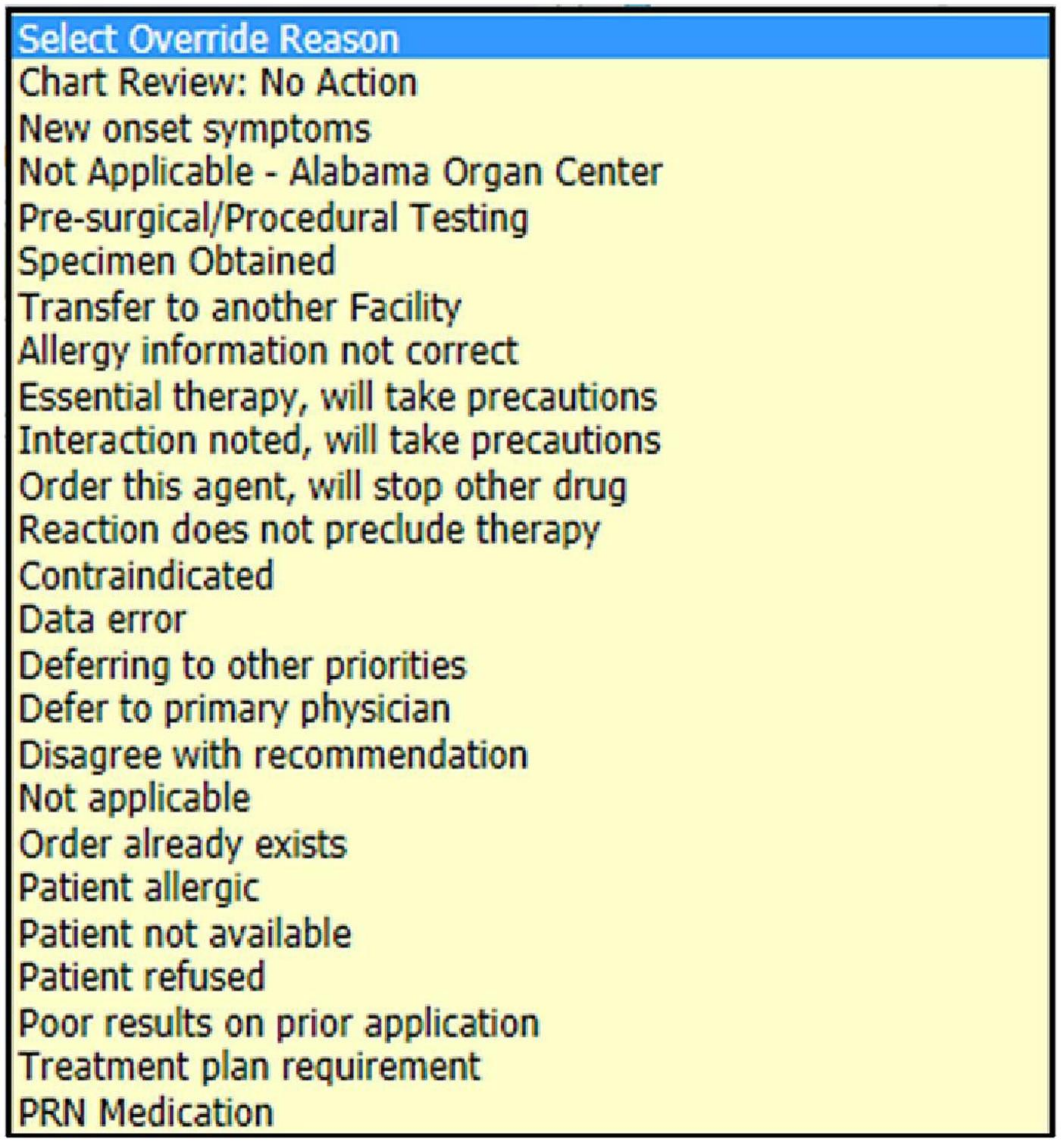
Override reasons showed for DAAs in EHR.

When a DAA is triggered, it delivers a notification message to the user and temporarily halts the order-entry process. This allows the provider the opportunity to review the alert and either agree with it or override it and select a reason for the override. We extracted the override status for each alert, categorizing it as either overridden or accepted, along with the reason provided for the override. Figure 1 illustrates an example of DAA presented to providers and Figure 2 illustrates override reasons in the EHR.

### Data source

Data were gathered from all DAA triggered to providers with prescribing authority, including physicians (attendings and residents) and advanced practice providers in the UAB Hospital’s EHR system during the 2023 calendar year. This dataset includes information from various inpatient settings, such as regular inpatient units, intensive care units, and the emergency department. The dataset included detailed information on allergy documentation, prior orders, and drug administration records.

All data collection and analysis were conducted with prior approval from the UAB Institutional Review Board, ensuring that all records were deidentified before analysis to protect patient confidentiality.

### Data analysis

We calculated the frequencies of DAAs and their corresponding override rates for all drug classes. Subsequent analyses focused on the triggering drug. Each alert listed one or more allergic reactions (eg, anaphylaxis) associated with the respective allergen that triggered the alert (eg, amoxicillin). Structured reactions were grouped and ranked by frequency and free-text reactions were normalized using natural language processing supplemented by human annotation by one of the authors (J.H.B.M.). Reactions were further classified as potentially immune-mediated or potentially life-threatening based on the classification developed by Topaz et al.^32^ We further analyzed alerts based on match type as described above. We compared the triggering drug against (1) the allergen documented for the patient, and (2) prior drug administrations of the same patient; the latter was used to determine if the patient had received the triggering drug in the past and whether override rate varied across patients who had or not had received the drug previously. Frequencies were calculated for each match type (definite, probable, or possible) and compared by alert status (accepted vs overridden). Overrides with no prior match or lacking evidence of prior drug administration (eg, patient has no documentation of prior administration of the triggering drug) were classified as “no match.” In a sub-analysis, we calculated the frequency of DAAs with prior definite match with future administration records from new orders up to the time of this writing (October 2025). Match types were compared using maximum-likelihood dichotomous logistic regression in STATA v.18.

## Results

### Characteristics of drug allergy alerts

**Figure 3.**
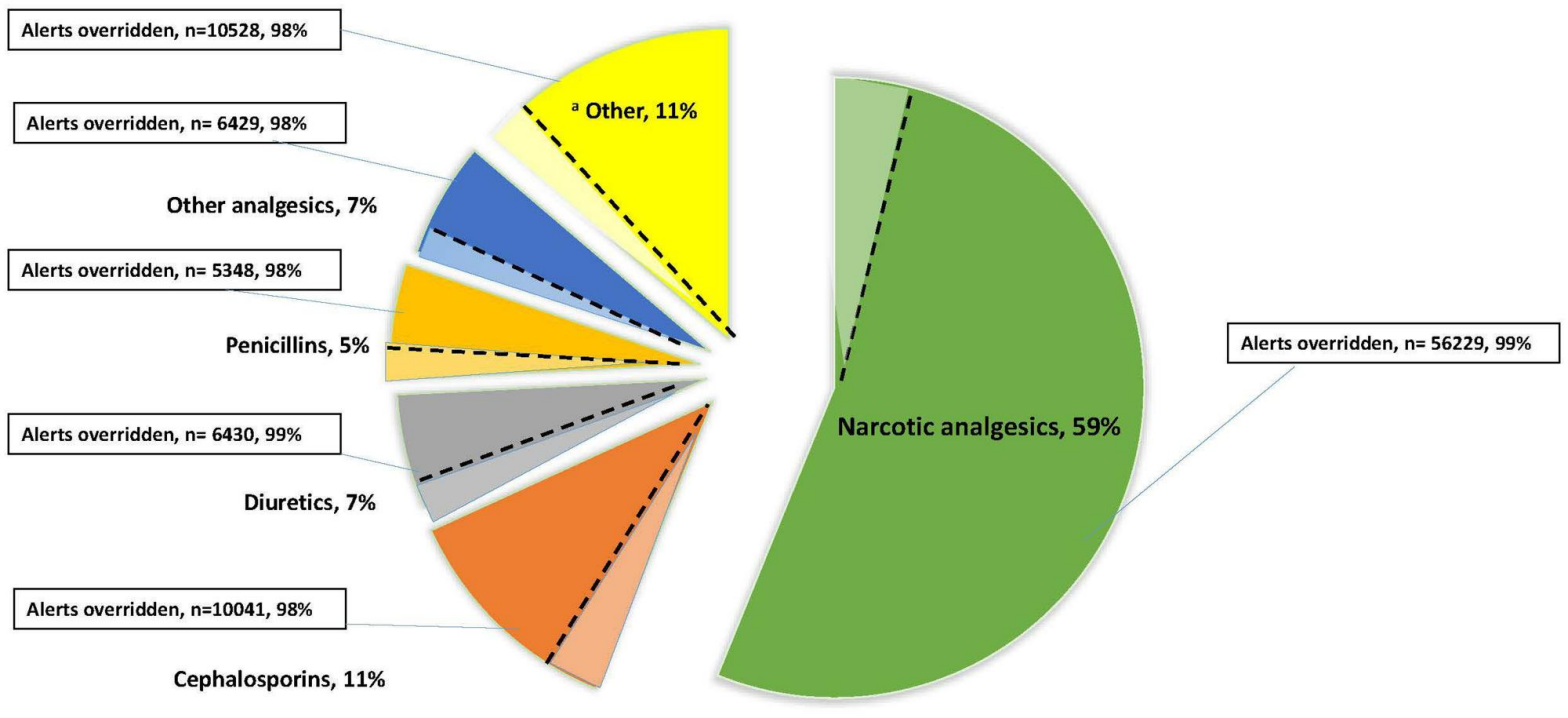
Distribution of DAAs overridden by drug class. Light colored portions of slices represent accepted alerts and dark colored portions represent overridden alerts. ^a^Other drug classes with volume of alerts < 1%.

A total of 101 492 DAAs were triggered in 2023, with an override rate of 98.9% (*n* = 100 418). The number of unique patients for the alerts fired was 9111, which results in an average of 11.1 alerts fired per patient (SD = 22.5). Among drug classes accounting for more than 1000 alerts, opioids had the highest volume of alerts (59%, *n* = 56 229) and were nearly 5 times as frequent as the next drug class (cephalosporins, 11%, *n* = 10 041). Diuretics had the highest override rate, (99.5%) followed by opioids (99.1%), penicillin (98.7%), and cephalosporins (97.8%) (Figure 3). The most common reasons for overriding DAAs were “Interaction noted, will take precautions” (42.8%), followed by “Chart Review: No Action” (29.1%), and “Essential therapy, will take precautions” (14.8%) (Table 1). The most frequent reactions in overridden DAAs were itching (21.3%), followed by rash (19.2%), and nausea (16.3%) (Table 2). The comparison of override rates across alert characteristics revealed a statistically significant difference in all comparisons. Yet, these differences did not indicate a clear override pattern as override rates were high in all cases. For instance, non-life-threatening reactions were more likely to be overridden than life-threatening reactions (OR = 1.37, 95% CI: 1.12-1.69, *P* = .002) despite having a slightly higher override rate (98.9% [*n* = 54 251] vs 98.5% [*n* = 7990]). Likewise, non-immune-mediated reactions were more likely to be overridden than immune-mediated reactions (OR = 1.43, 95% CI: 1.20-1.69, *P *< .001) with a moderately higher override rate (99.1% [*n* = 20 990] vs 98.8% [*n* = 41 251]).

**Table 1.** Frequency of override reasons.

Override reasons	Frequency, *n* (%)
Interaction noted, will take precautions	42 317 (42.8)
Chart Review: No action	28 782 (29.1)
Essential therapy, will take precautions	14 631 (14.8)
Order already exists	2583 (2.6)
Not applicable	2491 (2.5)
Treatment plan requirement	1229 (1.2)
New onset symptoms	529 (0.5)
Order this agent, will stop offer drug	478 (0.4)
Allergy information not correct	469 (0.4)

**Table 2.** Top 10 reactions for documented allergies of the alerts overridden.

Reactions	Frequency, *n* (%)	Potentially immune-mediated^a^	Potentially life-threatening^a^
Itching	13 240 (21.3)	Yes	No
Rash	11 956 (19.2)	Yes	No
Nausea	10 175 (16.3)	No	No
Vomiting	9023 (14.5)	No	No
Hives	8383 (13.5)	Yes	No
Swelling	4422 (7.1)	Yes	Yes
Anaphylaxis	1942 (3.1)	Yes	Yes
Headache	1474 (2.4)	No	No
Shortness of breath	1308 (2.1)	Yes	Yes
Altered mental status	319 (0.5)	No	Yes

a Classification of reactions as potentially immune mediated or potentially life-threatening was based on the classification developed by Topaz et al.^32^

### Drug-allergy match types

DAAs were more frequently triggered for probable match (65.8%), followed by possible match (24.5%), and only 9.7% for definite match. A significant difference was observed between the override status and match type across all comparisons. Likewise in the comparison across alert characteristics, although statistically significant, these differences did not indicate a clear override pattern, since overrides tend to be high in all scenarios. Specifically, when comparing the triggering drug to documented allergens, probable matches (*n* = 66 801) were more likely to be overridden than definite matches (*n* = 9806) (OR = 1.28, 95% CI: 1.06-1.56, *P *= .012) and possible match (OR = 1.33, 95% CI: 1.17-1.53, *P* = .001), despite having a slightly higher override rate (99% vs 98.7%, vs 98.7%). Possible (*n* = 24 885) and definite matches (*n* = 9806) had a similar override rate (98.7% vs 98.7%) without a significant difference in the logistic regression (OR = 1.04, 95% CI: 0.84-1.28, *P* = .73). When comparing the triggering drug with previously administered drugs, definite match alerts (*n* = 71 357) were more likely to be overridden than no match (*n* = 30 135) (OR = 1.18, 95% CI: 1.03-1.33, *P* = .013) with a slightly higher override rate (98.9% vs 98.8%) (Table 3). In over two-thirds (70%, *n* = 71 357) of DAAs the patient had receive the triggering drug in the past, of these, 74% (52% of all DAAs) the drug had been administered after the allergy had been documented in the patient’s record, and 79% (56% of all DAAs) the patient received the triggering drug in a new future order.

**Table 3.** Inpatient drug allergy-alert by match types.

**Match type** ^a^	**Alerts, *n* (%)** ^b^	**Overrides, *n* (%)** ^c^	Override rate
**Triggering drug vs allergen** ^d^			
Probable	66 801 (65.8)	66 163 (65.9)	99%
Definite	9806 (9.7)	9686 (9.6)	98.7%
Possible	24 885 (24.5)	24 569 (24.5)	98.7%
**Triggering drug vs prior administration** ^e^			
Definite	71 357 (70.3)	70 639 (70.3)	98.9%
No match	30 135 (29.7)	29 779 (29.7)	98.8%

a Potential match types between triggering drug and allergen/prior administration.

b Total drug alerts categorized by match type.

c Overridden drug alerts categorized by match type.

d Comparison between prescribed drug and drug documented as allergen.

e Comparison between prescribed drug and prior drug administered drugs.

Definite match: exact match between allergen and prescribed drug or it its main ingredient, eg, fentanyl—fentanyl.

Possible match: drug class of the allergen matches the drug class of the prescribed drug. eg, morphine-codeine.

Probable match: the potential cross-reactivity based on shared structural features or metabolic pathway, though not from the same drug class, eg, furosemide, and bactrim.

No match: No relationship found between the prescribed drug and allergen or prior administration.

## Discussion

While in our preliminary study^29^ we explored the feasibility of using prior drug administrations to identify false-positive DAAs using one high-volume drug class (opioids), in this study we expanded our analysis to all drug classes using recent DAA instances to increase external validity. We investigated the characteristics of DAAs that were overridden at our institution and examined several aspects of override rate using previous drug administrations. While several studies have explored factors associated with DAA overrides to better understand alert and override patterns, they have primarily focused on examining alert frequency, override rate, and appropriateness of overrides.^17^^,^^33^ Others have analyzed DAAs triggered and overridden based on match type,^34^ but their investigations were limited to comparing the triggering drug with documented allergens, overlooking previously administered drugs. While some researchers have assessed the appropriateness of DAA overrides by examining override reasons informed by providers in the alert dialog box (eg, pop-up window),^35^^,^^36^ others have confirmed through chart review whether the override resulted in actual medication administration,^20^ but have not examined whether previous administrations of the triggering drug occurred. Prior research has also highlighted instances where patients have previously received a triggering drug, as evidenced by prior administration records, but these investigations have not tested the association of prior administrations with override rate.^28^ We have previously tested this association but have focused on opioid DAAs only.^29^ To the best of our knowledge, this is the first study to examine not only the evidence of prior administration by match type but also their frequency based on override status for DAAs in all drug classes.

Our overall DAA override rate was 98.9%, which is slightly above the range of 44%-97% reported in a recent systematic review of DAA systems.^37^ We confirmed that opioid DAAs continue to be the most commonly triggered alert, aligning with previous similar examinations.^32^^,^^38^ While several studies have evaluated the appropriateness of DAA overrides based on prescribers’ informed override reasons,^33^ previous studies show that they not always represent a conscious decision because providers may override alerts without intentionally picking an appropriate reason.^29^ Informal discussions with physicians at our institution revealed that due to the high frequency of false-positive alerts, clinicians often anticipate when such alerts will occur and select a meaningless reason (eg, the option set as default). Further, this type of automatic response to CDS alerts, often referred to as “automatism,” has been reported in the literature^8^ and empirically observed.^39^

Our findings showed that the most frequently overridden allergy reactions align with those reported in other DAA override studies.^32^ Reactions like itching (21.3%), rash (19.2%), and nausea (16.3%), which are non-life-threatening but potentially immune-mediated, were overridden more often than severe reactions such as swelling (7.1%), anaphylaxis (3.1%), and shortness of breath (2.1%). Similarly, common and generally less serious non-immune-mediated reactions like vomiting (14.5%) and hives (13.5%) were more frequently overridden than potentially life-threatening ones such as altered mental status (0.5%), a pattern that is often observed in examinations of DAA overrides.^32^^,^^35^

Alerts triggered for definite matches were overridden more frequently than those for possible or probable matches, but the difference may not be clinically meaningful due to consistently high override rates in all situations. This aligns with previous DAA studies, which reported statistically significant results with uncertain clinical impact.^35^ However, the frequency of definite matches for prior administrations may have clinical significance, as research shows that clinicians are more likely to override DAAs when a patient has previously tolerated the medication, but previous reports have determined prior tolerance based on the provider-reported override reason alone.^34^ Further, prior research shows that excessive false-positive alerts may cause clinicians to overlook clinically relevant warnings with real potential to improve care decisions.^3^^,^^19^ In our organization, over two-thirds (70%) of DAA overrides had a definite match for prior administered drugs; of these, 74% (52% of all DAAs) had been administered after the allergy had already been documented in the patient’s record, and 79% (56% of all DAAs) the triggering drug was administered again in the same patient in the future, in other words, a prior DAA had already been overridden for the same drug and patient, followed by the administration of the drug in the past, and the patient continued to receive the drug in future orders post 2023. These findings indicate that 52%-70% of all DAAs can potentially be safely suppressed based on presumed prior tolerance of the triggering drug, especially since the vast majority of DAAs (90%) were triggered by a drug different than the one documented as allergen, which in many cases involves low-risk cross-sensitivity pairs such as synthetic and natural opioids.^29^^,^^40^^,^^41^ An alternative explanation is that our providers may override most interruptive alerts as a form of “automatism,” which has been empirically demonstrated in prior examinations of alert override,^8^ and is consistent with an overall override rate of interruptive alerts of 81.3% at our institution.^42^ This aspect, however, remains an underexplored area in CDS research, which hinders an objective assessment of the generalizability of our findings and the degree to which a meaningful alert suppression can be achieved without compromising patient safety.

Prior studies have attempted to enhance allergy documentation using manual and semi-automated approaches, but have achieved only marginal results.^25–27^ These interventions include nursing reconciliation pharmacist-driven interventions, and adding a link to update allergy documentation in the DAA dialog box, which rely on manual interventions that leave persistent documentation shortfalls, suggesting a need for further studies examining other approaches, including potentially automated solutions combining improved allergy records with more effective CDS logic.^33^^,^^37^^,^^43^

While our prior study had indicated that opioids are a major contributor to high DAA override rates,^29^ our current study supports a broad class-based alert suppression approach, as indicated by 70% (*n* = 71 357) of all DAA overrides with a prior administration of the triggering drug, of which 52% (*n* = 52 776) occurred after the allergy had already been documented. Thus, we hypothesize that in most cases the prescriber was aware that the patient had tolerated the prescribed drug in the past when the DAAs in our sample were triggered. This hypothesis warrants further investigation.

It is important to note that this approach is not restricted to DAAs, suggesting that the total number of alerts eligible for suppression across other types of drug alerts (eg, drug-dose, drug-drug interaction) could be significantly higher. Further investigation is needed to assess this potential, including confirmation that prior administrations were well-tolerated and did not result in ADEs, and that other types of alerts with specific scenarios (eg, changed patient status due to renal failure developed after drug administered) are accounted to ensure prior drug tolerance.

### Future research and mitigating alert fatigue

Manual and semi-automated methods like nursing reconciliation and CDS alerts improve allergy documentation but leave gaps, underscoring the need for more integrated, automated solutions. Machine learning (ML) techniques have been explored as a potential solution for reducing alert fatigue.^44^^,^^45^ However, these methods also face challenges, including limited generalizability due to training datasets from single organizations and automation bias caused by reliance on user responses to alerts, which may perpetuate suboptimal clinical decisions.^46^ More recently, large language models (LLMs) have been tested across multiple scenarios, including CDS optimizations,^47^ but such studies have not attempted to use patient-specific data determine alerts eligible for suppression, nor whether LLMs are an effective tool to determine drug tolerance by combining assessments of both structured (eg, antihistamine orders) and narrative data (eg, reactions documented in clinical notes) needed for this determination.

We have implemented a patient specific knowledge base,^48^ which contains drug tolerance assertions in the form of triples (subject-relationship-object) used to turn implicit statements about drug tolerance into explicit tolerance assertions based on prior drug administrations. This method could be further enhanced with rules identifying implicit indications of drug tolerance, such as repeated refills of a drug documented as allergen or multiple overrides for the same drug, supplemented with automatic screening of clinical notes using LLMs to determine the occurrence of ADEs following the drug administration.^48^ Some drugs such as vancomycin may produce a reaction in patients with prior tolerance.^49^ To minimize the risk of ADEs in these cases, safety filters can be added to the CDS logic to determine drug ingredients in which a prior administration warrants DAA suppression or not. Although this approach would not rule out the occurrence of reactions or guarantee the highest alert suppression rate possible, it can be used to make DAAs more informative, providing individualized drug tolerance assertions in interruptive alerts, or replacing interruptive alerts with context-aware passive alerts that do not interrupt order entry but allow the provider to act upon the alert if needed (eg, review prior tolerance history and decide whether the order should continue or not).

To advance the improvement of alert accuracy and clinician response to alerts, several challenges to modifying CDS logic remain due to concern over legal liability resulting from disabling alerts, even when they have been proven inefficient.^50^ Other challenges involve limited flexibility of the CDS infrastructure of widely adopted commercial EHRs, which limit parametrization of DAA filters to allergy reactions and their severity level, which are insufficient to determine drug tolerance; currently, this barrier cannot be overcome even with the use of prominent CDS standards such as CDS Hooks.^43^ Improving CDS design and accuracy will require collaborative relationship with EHR and CDS vendors, policy makes and standard organizations to enable third-party CDS solutions to augment the native logic in commercial EHRs, giving users the flexibility needed to decide which alerts to suppress and under what conditions, while preventing patient safety and vendor control over their products.

On balance, a patient-specific, standards-based approach has the potential to reduce clinically irrelevant DAAs and empower health care organizations to minimize alert burden while preserving safety through more effective CDS. Future work should investigate methods such as LLMs applied to narrative data coupled with structured data form the patient’s records (eg, antihistamine orders) to confirm or rule out tolerance assertions derived from drug administration records.

### Limitations

Our findings are based on retrospective data from one organization using a specific commercial EHR, which may not capture the context or decision-making nuances that may be observed in other organizations and EHRs. Results may not be generalizable to all healthcare settings, as variations in EHR systems, clinical workflows, and alert configurations could impact alert override behavior. However, most of our observations are consistent with prior studies on DAAs, increasing the likelihood of external validity.

The study does not account for confounders and predictors of behavior such as prescription of desensitization protocols, intentional acceptance of risk by clinicians, or changes in patient health status; yet, prior literature shows that such preventive measures are not consistently implemented.^51–53^ Factors such as prescribing patterns, and provider and patient characteristics were not included in our analysis and may be associated with override pattern. Our analysis assumes that prior drug administration without adverse events equates to drug tolerance, which might not always hold true (eg, delayed reactions, subtle symptoms, not documented reaction). On the other hand, we may also have misrepresented the true number of prior administrations in cases where an administration was documented during hospitalizations that occurred in another organization. Allergy documentation in EHRs may not always be accurate, complete, or up-to-date, leading to potential misclassification of alerts and tolerance assertions.

## Conclusion

In this retrospective observational study, we examined how frequently prior drug tolerance administration is observed in all DAAs overridden by providers with prescribing authority. Our findings suggest that a significant proportion of DAAs (70%) could potentially be suppressed based on prior drug administration, of these 52% are indicative of likely tolerated drugs as the they represent DAAs for which the triggering drug had been administered after the allergy had already been documented in the patient’s record. Further, in 79% of DAAs with previous administration record the patient received the triggering drug at least once again in the future (after 2023), which is an additional assertion of presumed drug tolerance. Future research should focus on examining strategies to confirm these assertions using data from the patients records and safely incorporate drug tolerance into the CDS logic to enable alert suppression without comprising patient safety.

## Data Availability

Structured data collected for this project can be stripped of identifiers and dates can be shifted to preserve subjects’ privacy, allowing our dataset to be made available along with the respective metadata to other researchers via collaboration systems such as GitHub.
